# Removal of calcium hydroxide from Weine Type II systems using photon-induced photoacoustic streaming, passive ultrasonic, and needle irrigation: a microcomputed tomography study

**DOI:** 10.1590/1678-775720160234

**Published:** 2016

**Authors:** Adam LLOYD, Geraldine NAVARRETE, Melissa Andreia MARCHESAN, David CLEMENT

**Affiliations:** 1 - University of Tennessee Health Science Center, College of Dentistry, Department of Endodontics, Memphis, TN, USA.; 2 - The University of Oklahoma, College of Dentistry, Oklahoma City, OK, USA.

**Keywords:** Endodontics, Lasers, Ultrasonics, Intracanal dressing, Irrigants

## Abstract

**Objective:**

This study compared the effectiveness of Er:YAG laser-activated irrigation (PIPS), passive ultrasonic irrigation (PUI) with EndoUltra and standard needle irrigation (SNI) in the removal of calcium hydroxide [Ca(OH)_2_] from the mesial roots of Weine Type II mandibular molars.

**Material and Methods:**

Thirty mandibular molars were screened by µCT for the presence of mesial roots with complex intra-canal anatomy and a common apical foramen. The teeth were enlarged to a standardized 25/.06 preparation and filled with Ca(OH)_2_ paste. Specimens were divided into three groups (n=10) according to the technique used for Ca(OH)_2_ removal: PIPS, at 15 Hz and 20 mJ using a 9 mm long, 600 µm diameter tip; PUI using a 15/.02 tip; and SNI (30 Ga. side-vented needle). Equal volumes of 8.25% NaOCl and 17% EDTA were used in all groups. µCT was used to measure the initial amount of Ca(OH)_2_ present and to assess the residual volume of Ca(OH)_2_ following each irrigation protocol. Data were analyzed using Tukey HSD and Kruskal-Wallis tests (α=5%).

**Results:**

The mean volume of Ca(OH)_2_ before removal was significantly higher in the coronal third than in the middle and apical third (p<0.001). Ca(OH)_2_ was similarly removed from the coronal and middle thirds with the three methods used (p>0.05). PIPS (median 0%; IQR: 0-0) showed significant higher Ca(OH)_2_ removal in the apical third than PUI (median 100%, IQR: 85-100) and SNI (median 47%; IQR: 16-72) (p<0.001).

**Conclusions:**

PIPS laser-activation was more effective for the removal of Ca(OH)_2_ from mesial roots of mandibular molars with Weine Type II canal configurations than PUI with EndoUltra and SNI.

## INTRODUCTION

Intracanal medicaments have been used to further reduce the bacterial load of the root canal system that chemomechanical debridement may not remove^20^. Among these, calcium hydroxide [Ca(OH)_2_] has been the most widely accepted intracanal medicament because of its high pH (12.5) and its ability to significantly reduce the bioburden and endodotoxin concentrations in teeth with apical periodontitis^1^ ^,^ ^25^. Concerns have been raised about the potential interactions of the remaining Ca(OH)_2_ and endodontic sealers. Margelos, et al.^19^ (1997) demonstrated incomplete setting and increased brittleness of zinc oxide-eugenol based sealers. Barbizam, et al.^3^ (2008) reported diminished adhesion of Epiphany sealer to root canal walls. Kim and Kim^11^ (2002) reported that the potential for Ca(OH)_2_ to dissolve in water could influence the long term leakage of root canal obturation. Hence, prior to obturation, intracanal medicaments must be removed from the root canal system to allow the penetration of root canal sealer into canal intricacies and dentinal tubules leading to a hermetic seal^15^.

Different techniques have been proposed for the removal of Ca(OH)_2_ from the root canal system, including a combination of irrigating solutions and devices^6^ ^,^ ^18^ ^,^ ^23^. The most frequently described technique includes a combination of irrigation and recapitulation of the master apical file^23^. In this instance, only the Ca(OH)_2_ in the main canal will be disrupted with the file while persisting in system extensions or irregularities relying entirely on irrigation for the removal of Ca(OH)_2_ from these regions. While some investigators have shown a combination of ethylenediaminetetraacetic acid (EDTA) and sodium hypochlorite (NaOCl) irrigants completely remove Ca(OH)_2_ from single systems^6^, others report that complete removal of medicaments from such teeth is unlikely^7^. Furthermore, they suggest depth of needle penetration plays a significant role in the removal process^7^.

The effectiveness of irrigation depends on the chemical action of the irrigant and the ability to bring the irrigant in contact with the complex structures within the root canal system^22^ ^,^ ^26^. Energizing irrigants with activated devices has been used to enhance fluid interchange when compared with needle irrigation. Passive ultrasonics have demonstrated enhanced irrigation through the creation of eddy currents and microstreaming along the instrument shaft and improved Ca(OH)_2_ removal^2^ ^,^ ^18^. A recent study evaluated the effect of the GentleWave system (Sonendo Inc, Laguna Hills, California, USA) in the removal of Ca(OH)_2_ from mandibular molars with two separate mesial canals^18^. The investigators found that this treatment was able to render mesial canals free of Ca(OH)_2_ when compared with passive ultrasonic irrigation (PUI) and standard needle irrigation (SNI). Although this system shows encouraging results, there are drawbacks to its clinical use, including the difficulty in attaching a customized disposable handpiece to individual teeth for stabilization creating a closed system for delivery of the multisonic stream of irrigants. Promising results for the removal of Ca(OH)_2_ have also been shown using an Erbium:Yttrium-Aluminum-Garnet (Er:YAG) laser coupled with a short radial-stripped tip in a technique known as photon-induced photoacoustic streaming (PIPS; Fidelis; Fotona, Ljubljana, Slovenia). Arslan, et al.^2^ (2015) demonstrated that PIPS irrigation provided complete removal of Ca(OH)_2_ from artificial grooves in single-rooted teeth when compared with ultrasonic, sonic, and SNI techniques, which did not.

Multiple investigations have examined Ca(OH)_2_ removal from non-complex single-rooted teeth that may present misleading information not representative of the complex anatomy encountered by endodontists^2^ ^,^ ^6^ ^,^ ^23^. Recent microcomputed tomographic (µCT) studies on the anatomical complexities of the mesial roots of mandibular molars have shown a prevalence of isthmuses of approximately 80% between 3-6 mm from the apex^9^ ^,^ ^26^. These anatomical structures can retain debris from instrumentation^8^ ^,^ ^21^; microbes^5^; and Ca(OH)_2_ ^17^ ^,^ ^18^.

The use of µCT imaging to volumetrically assess the removal of Ca(OH)_2_ allows for a three dimensional quantitative evaluation based on gray values with higher sensitivity than sectioning techniques^28^. The purpose of this study was to use μCT imaging to assess the efficiency of PIPS laser-activated irrigation, a PUI with EndoUltra and SNI in the removal of Ca(OH)_2_ from the mesial roots of Weine Type II mandibular molars with complex anatomy.

## MATERIAL AND METHODS

Tooth selection followed protocol approval from an Institutional Review Board with no patient health identifiers associated with the obtained samples (14-03540-XM). Mandibular molars were screened for the presence of isthmuses and Weine Type II canal configurations (two orifices - two canals - one apical foramen) using a high-resolution µCT scanning system (ACTIS BIR 150/130, Varian Medical Systems, Palo Alto, California, USA). The images were acquired at 75 kV and 100 µA through 360° of rotation around the vertical axis resulting in a cross-sectional pixel size of approximately 30 µm. Each backscatter projection had a 16-bit addressable 1,024×1,024 area and was used to create a volume-rendered representation (VG Studio Max 2.3; Volume Graphics GmbH, Heidelberg, Germany). Thirty teeth were selected and the distal root of each sample was sectioned with a diamond disc just below the orifice and sealed with unfilled resin. Samples were individually mounted in a Teflon mold to allow precise positioning in a jig on the µCT stage.

All endodontic procedures were performed under the clinical microscope at ×14 magnification (OPMI Pico, Carl Zeiss Meditec Inc., Jena, Germany) by a board-certified endodontist with 20 years of clinical experience. Canals were instrumented in a Crown-Down manner, briefly: #6 down to #3 Gates-Glidden drills (Dentsply Maillefer, Ballaigues, Switzerland), followed by 25/.12 and 25/.10 K3 Orifice Openers (SybronEndo, Orange, California, USA), and by #30/.06, 35/.04, and 25/.06 Profile Vortex (Dentsply Tulsa Dental Specialties, Tulsa, Oklahoma, USA) instruments. The apical preparation was standardized to a 25/.06 at 0.5 mm from the apical foramen and apical patency was verified with a #10 K-file. Postinstrumentation irrigation was performed with alternating 8.25% NaOCl (The Clorox Co, Oakland, California, USA) and 17% EDTA (Roth Drug Co, Chicago, Illinois, USA) with PIPS laser-activated irrigation at a wavelength of 2,940 nm in 30 s exposure intervals at 15 Hz and 20 mJ. A 9 mm long, 600 µm diameter quartz tip, with the polyamide tip stripped back 3 mm was used. All samples were dried with sterile paper points and a premixed calcium hydroxide/barium sulfate paste (Ultracal XS, Ultradent Products Inc, South Jordan, Utah, USA) was injected into the mesial canals using a 30 Ga NaviTip (Ultradent). A Lentulo spiral filler (#2, Miltex, York, Pa., USA) was used 1 mm short of the binding point in a slow-speed handpiece until Ca(OH)_2_ was visualized at the apical foramen^27^. A cotton pellet was placed on the floor of the pulp chamber and the access cavity was sealed (Cavit, 3M ESPE, Seefeld, Germany). A proximal radiograph was taken to confirm the intracanal Ca(OH)_2_ length and density. The apices of all specimens were sealed with sticky wax. The specimens were stored at 37°C in 100% humidity for seven days and a second µCT scan was performed to quantify the percentage volume of Ca(OH)_2_ occupying the root canal system.

The specimens were accessed again and a size 25 Flex-O-file (Dentsply Maillefer, Ballaigues, Switzerland) along with 3 mL of NaOCl was inserted into each canal for the working length to loosen the Ca(OH)_2_ ^14^ ^,^ ^23^. The specimens were then randomly distributed into three experimental groups (n=10):

### Group 1

Laser-activated irrigation using an Er:YAG laser and a PIPS tip was performed according to the manufacturer’s instructions. The tip was placed into the access cavity only and activated with each of the following irrigating solutions as they were introduced into the chamber with a 28 Ga side-vented irrigation needle:

Step 1: Three 30 s cycles of 6 mL/interval of 8.25% NaOCl interrupted by a 30 s wait between each cycle

Step 2: 30 s cycle of 6 mL of water

Step 3: 30 s cycle of 6 mL of 17% EDTA

Step 4: 30 s cycle of 6 mL of water

### Group 2

The PUI with EndoUltra protocol was the same as for PIPS following the steps previously described. However, PUI was delivered using nickel-titanium activator tips (15/.02) in a cordless ultrasonic handpiece (EndoUltra, Vista, Racine, Wisconsin, USA) and activated as far apically as achievable without binding in 2 mm amplitude motions.

### Group 3

SNI was conducted with a 30 Ga side-vented needle delivering 18 mL of 8.25% NaOCl over a period of 90 s at a distance of 2 mm close to the working length with 2 mm amplitude movements, followed by 6 mL of water, 6 mL of 17% EDTA delivered over 30 s, and a final rinse of 6 mL of water.

The total volume of irrigation was the same in all experimental groups.

A final μCT scan was performed following irrigation. Three-dimensional (3D) volumes were automatically generated from thresholding and region growing based on grey values, separating Ca(OH)_2_ from dentin. The unchanged outer root surface of the second and final scans allowed precision alignment of both overlaid 3D datasets to subvoxel accuracy (VG Studio Max 2.3, Volume Graphics GmbH, Heidelberg, Germany). Total Ca(OH)_2_ volume from each specimen was derived from opaque (bright) voxels within the confines of the radiopaque canal walls. Each specimen was measured from apical foramen to orifice and equally divided into coronal, middle, and apical thirds. Regions of interest for each third were created based on slice position and volumes recorded.

The images were qualitatively evaluated in a double-blind manner by two independent precalibrated evaluators to classify the removal of Ca(OH)_2_ from the isthmus area. A four-level score system was employed:

1= Clean isthmus, no Ca(OH)_2_.

2= Clusters of Ca(OH)_2_ filling ≤50% of the volume.

3= Clusters of Ca(OH)_2_ filling >50% of the volume.

4= Isthmus completely filled with Ca(OH)_2_.

The Shapiro-Wilk test was used to assess data normality. The mean volume of Ca(OH)_2_ *per* third before removal had a normal distribution and data were compared with one way-ANOVA and Tukey HSD tests. Kruskal-Wallis one way-ANOVA test was used to compare the mean percentage volume of Ca(OH)_2_ remaining after the different irrigation techniques. The specific pattern of differences between the interaction root canal thirds and irrigation techniques was demonstrated by Tukey’s HSD pairwise tests. The level of significance was set at α<0.05. The Kappa coefficient was used to determine the interobserver reproducibility for the removal of Ca(OH)_2_ from the isthmus area. Statistical analysis was performed using SigmaPlot 13.0 (Systat, San Jose, California, USA).

## RESULTS

The mean Ca(OH)_2_ volume before removal was 9.21±2.19, 9.69±2.34, and 10.83±2.50 mm^3^ for SNI, PIPS, and PUI respectively. The mean volume of Ca(OH)_2_ before removal was significantly higher in the coronal third than in the middle and apical third, and the middle was higher than the apical third (p<0.001).

**Figure 1:**
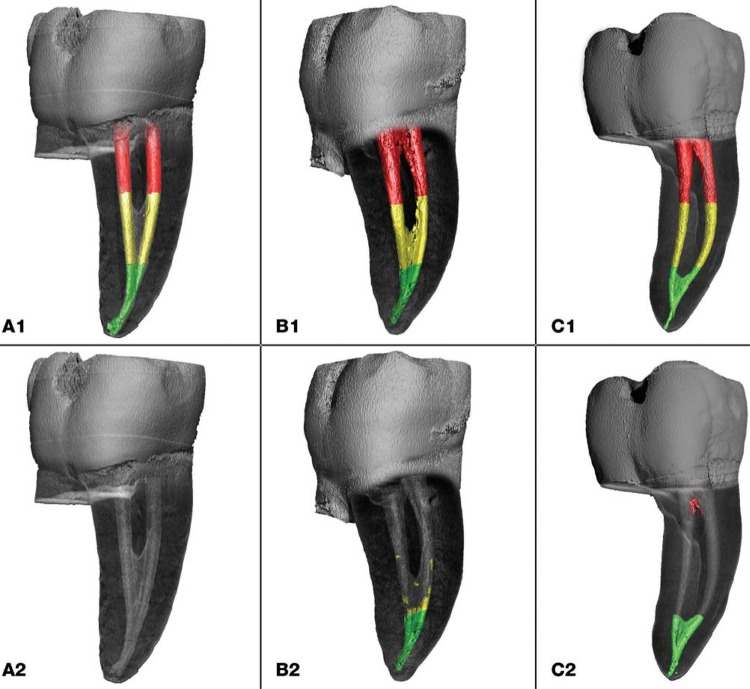
Three-dimensional reconstructions of mesial roots of mandibular molars with Weine Type II canal configurations illustrating remaining Ca(OH)2 in the different canal thirds (coronal – red; middle – yellow; apical – green) before (A1–C1) and after irrigation protocols (A2) PIPS, (B2) PUI, (C2) SNI.

The volume of Ca(OH)_2_ remaining in the mesial roots of Weine Type II mandibular molars was significantly different for the studied irrigation techniques (p<0.0001) (Figure 1). PUI (median 8.33%) and SNI (median 4.78%) showed the highest amounts of remaining Ca(OH)_2_ when compared with PIPS (median 0.00%), which showed the lowest (p<0.01). None of the specimens in the PUI group were completely free of Ca(OH)_2_, with the apical third showing the highest percentage of remaining Ca(OH)_2_ (median 100%). SNI showed a median of 47% of remaining Ca(OH)_2_ in the apical third and no differences between the thirds (p>0.05). PIPS showed a median of 0% of remaining Ca(OH)_2_ and no differences between the evaluated thirds (p>0.05). The interaction between the irrigation methods and the root canal thirds showed that all irrigation methods used were statistically similar in removing Ca(OH)_2_ from the coronal and middle thirds (Table 1). However, PIPS showed statistically significant higher removal of Ca(OH)_2_ in the apical third when compared with both PUI and SNI (p<0.001).

**Table 1:** Median (interquartile range) percentage of remaining Ca(OH)2 after irrigation with different techniques for canal thirds

	Coronal	Middle	Apical
PIPS	0% (0%, 0%)^aA^	0% (0%, 0%)^aA^	0% (0%, 0%)^aA^
PUI	0% (0%, 0%)^aA^	0.50% (0%, 5.82%)^aA^	100% (85%, 100%)^bB^
SNI	0.073% (0%, 0.416%)^aA^	1.7% (0.8%, 4.5%)^aA^	47% (16%, 72%)^aB^

Lowercase superscript letters indicate homogeneous subsets between canal thirds

Uppercase superscript letters indicate homogeneous subsets between methods

The interobserver Kappa coefficient was 0.89 for the removal of Ca(OH)_2_ from the isthmus area. A significant difference was found for PIPS (p*<*0.05) when compared with SNI and PUI, which were statistically similar (p>0.05), whereas higher scores, which imply more Ca(OH)_2_ remaining in the isthmus area, were found for SNI and PUI (Table 2).

**Table 2:** Distribution of scores for the Ca(OH)2 removal from the isthmus area for different irrigation protocols

Groups	Scores for Ca(OH)_2_ removal from the isthmus area			
	#1	#2	#3	#4
PIPS	9	1	0	0
PUI	2	5	3	0
SNI	2	3	5	0

## DISCUSSION

In the present study, all specimens were irrigated with PIPS prior to the placement of Ca(OH)_2_. PIPS has demonstrated the ability to remove debris from complex anatomy through high-velocity fluid interchange through expanding and collapsing cavitational bubbles along the root canal system^12^ ^,^ ^16^. ^s^ A pilot study showed that PIPS irrigation allowed for better penetration of the intracanal medicament into isthmuses and interconnections between main canals for the type of teeth selected for this study.

This is the first qualitative investigation to examine remaining Ca(OH)_2_ in the isthmus area, which showed similar findings observed for the volumes of Ca(OH)_2_ in the different thirds. The highest amount of remaining Ca(OH)_2_ occurred with SNI and PUI. The rationale for such occurrence is the surface tension barrier created in the apical part of the canal that does not allow adequate flow into these complex non-separated areas^18^.

Irrigation with the recently marketed PUI device showed an overall 8.33% of Ca(OH)_2_ remaining in the root canal system, with no removal of medicament from the apical third. The isthmus area showed clusters of Ca(OH)_2_ filling less than 50% of the volume. We believe this is due to the inability of the EndoUltra tip to reach beyond the buccal-lingual curvature in spite of the 25/.06 apical enlargement. The tips of the EndoUltra activator are a smooth 15/.02 wire, claimed by the manufacturer to be constructed from nickel-titanium. Manufacturer’s instructions recommend tip placement at 2 mm from the canal terminus. In our study, the tip was placed in the canal as far apically as achievable without binding to move freely and improve the irrigant flow dynamics in the apical third of the root canal system^13^. This is the first investigation of the effectiveness of EndoUltra as an adjunct to irrigation in the literature. EndoUltra did not appear to have sufficient energy to generate the acoustic streaming and hydrodynamic shear stress propagation to eliminate Ca(OH)_2_ from the apical third. This is in contrast to other PUI with EndoUltra irrigation devices that have shown more effective removal of Ca(OH)_2_ when compared with SNI^18^ ^,^ ^24^.

For the SNI group, the tip of the needle was placed at a distance 2 mm close to the working length without binding. A 30 Ga needle has an external diameter of 0.31 mm, which is equivalent to the dimensions of a canal prepared to a 25/.06 at 1.0 mm less than the working length. Fluid dynamics in such circumstances only allows fluid interchange 0.75 mm from the tip of the needle^4^ and explains the resulting 47% of Ca(OH)_2_ remaining in the apical third.

Unlike SNI and PUI, the PIPS tip remains in the access cavity providing, expanding, and collapsing cavitational bubbles that progress as shear forces along the canal walls^12^. Our results showed no remaining Ca(OH)_2_ throughout the root canal system in mesial roots of mandibular molars with Weine Type II canal anatomy and isthmuses. Even the most apical area of the root canal, which normally imposes increased irrigation difficulties^10^, was free of Ca(OH)_2_. The increased average fluid velocity from the middle to apical third may have contributed to these results^12^. Additionally, samples tested were Weine Type II canal systems that allow fluid interchange to occur in a circular pattern between the mesio-buccal and mesio-lingual canals. The enhanced fluid movement and high velocity shear stresses could have resulted from the inherent canal anatomy, potentiating Ca(OH)_2_ removal from the apical third.

Under the conditions of the present study, the use of PIPS laser-activated irrigation demonstrated consistent removal of Ca(OH)_2_ from the entire canal system of mandibular molars with Weine Type II canal configurations, including the most complex isthmus areas found in the apical half. The new PUI and SNI did not consistently remove Ca(OH)_2_ from the canal system. The efficacy of PIPS irrigation in removing Ca(OH)_2_ from mandibular molars with different canal configurations should be investigated in future studies.

## CONCLUSIONS

Within the limitations of this study, our findings suggest that PIPS laser-activation was more effective for the removal of Ca(OH)_2_ from mesial roots of mandibular molars with Weine Type II canal configurations than PUI with EndoUltra and SNI.
